# Fugitive Iodism

**Published:** 1888-09

**Authors:** 


### FUGITIVE IODISM ; ŒDEMA OF EYELIDS.

Dr. F. Hewkley, in the *British Medical Journal*, says, W. J. H., æt. 30, on May 7, complained of frontal neuralgic headache, which had varied in intensity during the preceding ten days. He could not bring to mind having had headache before, but had been, during several years, inconvenienced by an irritable catarrhal condition of his fauces and naso-pharynx. Having applied a chloroform preparation of aconite root with a camel-hair pencil horizontally to the forehead from side to side, a full inch above the eyebrows, I prescribed as follows: R. Liq. ferri iodidi, 1½ drachm; aq., 5½ ounces; ½ ounce ter. Soon after the second dose, the patient felt a severe cold had come upon him quite suddenly—sneezing, a very copious and steady flow of watery fluid from the left nostril, sufficient to saturate two handkerchiefs. This ceased in one hour as abruptly as it had occurred. Coincident with its cessation a puffiness of the eyelids on the corresponding side ensued ; this increased, and on retiring the patient's lids were completely closed. He slept well, and the morning following, his eye presented the appearance of having been stung by an insect, as described by Dr. Tom Robinson. The left eyelids, both upper and lower, were equally tumid with a semi-transparent rosy fullness, which the patient said was less than on retiring the night before. There was neither subconjunctiva œdema nor any discharge from between the lids. The conjunctiva presented a diffuse pinkness. Over both cheeks, but nowhere else on the body, was an urticarial erythema, fainter on the left than on the right cheek. He felt well, his headache was gone. On May 10, the phenomena had completely vanished.

REMARKS.—Here is seen a person with a decided catarrhal dyscrasia suddenly attacked with fugitive œdema of the eyelids sequent upon a free catarrhal outpour from the nasal mucous membrane. So far, the symptoms being limited strictly to the one side lends weight to the idea that an extension of the same catarrhal process into the neighboring loose connective tissue, though this was not related by immediate continuity, had occurred. Another view may be taken—that the transient œdema of the lids was associated with an impediment in the course of the efferent lymphatic circulation occasioned by the turgidity of the nasal mucous membrane.

